# Exploring the Recognition Mechanism of Surfactant–Cyclodextrin Complex Formation: Insights from SPR Studies on Temperature and Ionic Liquid Influence

**DOI:** 10.1021/acs.jpcb.4c04516

**Published:** 2024-09-20

**Authors:** Isabela Araujo Marques, Hauster Maximiler Campos de Paula, Camilla Fonseca Silva, Clebio Soares Nascimento Jr, Yara Luiza Coelho, Ana Clarissa dos Santos Pires, Luis Henrique Mendes da Silva

**Affiliations:** † Advanced Thermokinetics of Molecular Systems (ATOMS) Group, Chemistry Department, Federal University of Viçosa, Viçosa-MG 36570-000, Brazil; ‡ Theoretical and Computational Chemistry (LQTC) Laboratory, Department of Natural Sciences (DCNAT), Federal University of São João Del Rei, Dom Bosco Campus, São João Del Rei-MG 36301-160, Brazil; § Colloid Chemistry Group, Chemistry Institute, Federal University of Alfenas, Alfenas-MG 37130-000, Brazil; ∥ Applied Molecular Thermodynamic (THERMA), Food Technology Department, Federal University of Viçosa, Viçosa-MG 36570-000, Brazil

## Abstract

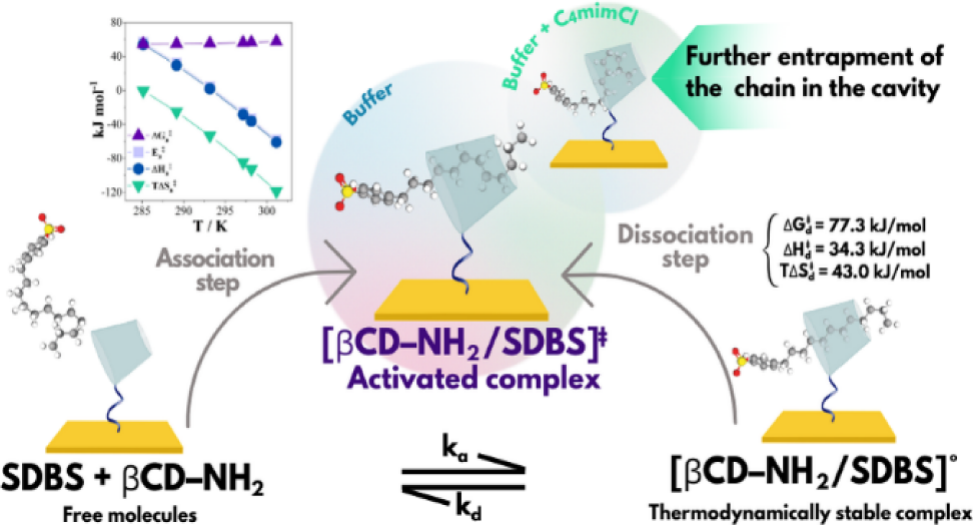

This study examines the kinetics and thermodynamics of the inclusion complex (IC) formation between sodium dodecylbenzenesulfonate (SDBS) and amine-modified β-cyclodextrin (βCD–NH_2_) using surface plasmon resonance (SPR) and theoretical analysis. We determined a binding constant of 10^3^ L mol^–1^ for the thermodynamically stable complex ([βCD–NH_2_/SDBS]°) within the temperature range of 285.2–301.2 K. The thermodynamic analysis revealed a transition from entropy-driven to enthalpy-driven behavior with increasing temperature. The rate constant for IC formation was approximately 10^2^ M^–1^ s^–1^, with the residence time decreasing from 14.08 s at 285.2 K to 6.13 s at 301.2 K. We observed the formation of an activated complex ([βCD–NH_2_/SDBS]^‡^), with energetic parameters indicating temperature dependence. At 285.2 K, the activated enthalpy change was positive, while at 301.2 K, it was negative. The dissociation energetic parameters remained temperature-independent. Additionally, increasing concentrations of the ionic liquid 1-butyl-3-methylimidazolium chloride influenced the SDBS tail’s conformation and penetration into the βCD–NH_2_ cavity at the activated state. These findings provide insights into the complexation mechanism and the effects of the temperature and ionic liquids on IC formation.

## 1 Introduction

Research on mixed cyclodextrin–surfactant systems remains highly active, encompassing a wide range of studies from the modulation of surfactant properties (e.g., critical micellar concentration)^1,2^ to the formation of self-assembly structures for applications, such as soil remediation,^3^ formation of multiple fluorescent emission color systems,^4^ control of calcium carbonate phases and morphology,^5^ and nanoencapsulation of food bioactives.^6^ Beyond these practical applications, ICs based on cyclodextrin–surfactant interactions serve as valuable models for studying the dynamics of supramolecular systems.

Supramolecular systems, including ICs, exhibit diverse behaviors under different environmental conditions, such as pH, temperature, and cosolutes. The ability to manipulate and tailor these systems as needed stems from a comprehensive understanding and modulation of the noncovalent interactions.^7^ In addition to intermolecular cohesion between host and guest molecules (e.g., hydrogen bonding, van der Waals forces, and electrostatic interactions), solvent effects and conformational fluctuations play pivotal roles in complex formation.^8^ The interplay of these weak forces allows the complexes to adopt various structures simultaneously, rendering the systems dynamic. Consequently, the functions exhibited by these systems result from a combination of these different states, not just the equilibrium product.^9^

While equilibrium studies provide valuable insights into supramolecular systems, they offer only a snapshot of the complexes after their formation.^10^ For example, extensive investigations into the thermodynamics of surfactant inclusion in β-cyclodextrin (βCD) have led to several key conclusions:^11−16^ (i) single-tailed surfactants (with tails up to 14 carbons) typically form 1:1 ICs with equilibrium constants that increase with the hydrophobicity of the tail; (ii) for ionic surfactants, the headgroup and counterion have minimal effects on the stability of the complexes formed; and (iii) the complexation process generally involves negative enthalpy changes due to intermolecular interactions, while the entropy change depends on the nature of the surfactant and cyclodextrin. However, to fully understand the complexation process, several questions require further investigation: (i) What are the energetic parameters for forming activated complexes in surfactant–cyclodextrin ICs? (ii) How does the temperature influence the kinetics of surfactant–cyclodextrin IC formation? Moreover, (iii) what is the effect of cosolutes on the recognition mechanism?

To address these questions, we conducted a study on the inclusion of the anionic surfactant sodium dodecylbenzenesulfonate (SDBS) in βCD in the absence or presence of the ionic liquid (IL) 1-butyl-3-methylimidazolium chloride (C_4_mimCl), from both kinetic and thermodynamic perspectives. In previous studies, Palepu et al., as well as Valente et al., utilized electrical conductivity measurements to establish the binding constants (1.5 × 10^4^ L mol^−117^ and 5.96 × 10^3^ L mol^–1^,^18^ respectively). Furthermore, they confirmed a 1:1 stoichiometry associated with the formation of the βCD/SDBS complex. Palepu et al. compared their results with those of other surfactants with dodecyl tails, highlighting the stronger binding of SDBS due to the benzene group. Liu et al. employed an isothermal titration calorimetry to investigate the inclusion process, obtaining a binding constant of 4.98 × 10^3^ L mol^–1^ and observing an enthalpy-driven process.^19^ Furthermore, Ogoshi et al. conducted a structural study using 2D ROESY measurements, revealing the partial inclusion of the dodecyl chain of SDBS into βCD.^20^

While these studies offer valuable insights into the βCD/SDBS system, there is a notable gap in the literature regarding a comprehensive understanding of the complexation mechanism, particularly its dynamic aspects. To address this gap, we employed surface plasmon resonance (SPR) to investigate the recognition mechanism involved in, including SDBS in βCD, complemented by theoretical studies. Specifically, our study aimed to elucidate this mechanism by evaluating the impact of temperature and the presence of C_4_mimCl.

## 2 Experimental Section

### 2.1 Materials

Sodium dodecylbenzenesulfonate (technical degree), β-cyclodextrin (>97%), 1-butyl-3-methylimidazolium chloride (>98%), sodium acetate (analytical grade), acetic acid (analytical grade), iodine, triphenylphosphine, imidazole, and ethylenediamine were purchased from Sigma-Aldrich (St. Louis, MO, USA). Research-grade CM5 sensor chips and the coupling reagents (*N*-ethyl-*N*′-(dimethylaminopropyl)carbodiimide hydrochloride (EDC), *N*-hydroxysuccinimide (NHS), and HBS-EP buffer (0.01 M HEPES pH 7.4, 0.15 M NaCl, and 0.005%v/v surfactant P20)) were purchased from GE Healthcare (Pittsburgh, PA, USA).

### 2.2 Synthesis of the Functionalized βCD

Before the SPR analysis was performed, βCD was functionalized with an amine group to allow the immobilization on the carboxymethyl dextran-modified gold surface of the CM5 sensor chip. The functionalization procedure was performed according to Hudson et al. Initially, βCD was iodinated to produce mono-(6-iodo-6-deoxy)-beta-cyclodextrin. This compound was then used to synthesize mono-(6-ethanediamine-6-deoxy)-beta-cyclodextrin (βCD–NH_2_).^21^

### 2.3 Surface Plasmon Resonance

The kinetics and thermodynamics of the inclusion of SDBS in βCD–NH_2_ were accessed through SPR analyses by using a Biacore X100 instrument (GE Healthcare, Pittsburgh, PA, USA) equipped with an automatic flow injection system. First, the carboxylic groups on the chip’s surface were activated by passing an EDC/NHS (0.4 M/0.1 M) mixture for 7 min. Then, the immobilization of the ligand was carried out according to the amine coupling method^22^ by injecting a βCD–NH_2_ solution (2.6 × 10^–5^ M, sodium acetate buffer (10 mM), pH 4) into the sample flow cell at a flow rate of 10 μL min^–1^ for 7 min, at 298.2 K. This procedure resulted in a low-density immobilization of βCD–NH_2_ to reduce mass transport and crowding phenomena.^23^ After immobilization, the excess of activated carboxyl groups was then blocked with ethanolamine for another 7 min. To discount systematic noise and Biacore drift, a reference flow cell was prepared as described above but without the immobilization of βCD-NH_2_.

For the interaction experiments, SBDS solutions (50–260 μM), prepared in HBS-EP buffer (pH 7.4) in the absence or presence of C_4_mimCl (2.5–10 μM), were injected in the flowing system to increase their concentration over the sample and reference surfaces. The experiments were carried out at temperatures ranging from 285.2 to 301.2 K. The buffer at pH 7.4 was injected before each SDBS/βCD–NH_2_ binding cycle to obtain the baseline.

### 2.4 Theoretical Studies

A sequential theoretical methodology was adopted to investigate the ICs between βCD–NH_2_ and SDBS or C_4_mim^+^. The process involved two stages:

1) Semiempirical optimization: PM3,^24^ a semiempirical method, was initially employed to optimize of both βCD–NH_2_/SDBS and βCD–NH_2_/C_4_mim^+^ complexes geometry. This approach provided a computationally efficient initial guess for the subsequent *ab initio* calculations.
2) Density functional theory (DFT) calculations: Following the PM3 preoptimization, single-point calculations were performed using DFT. The B97D functional,^25^ known for its accuracy in dispersion interactions, was chosen for this purpose. Pople’s standard 6-31G(d,p) basis set^26^ was utilized, offering a well-established balance between accuracy and computational cost.

Subsequently, the thermodynamic properties (enthalpy and Gibbs free energy changes) were computed for the complexes in an aqueous medium. The solvation model based on density (SMD)^27^ was employed to account for the implicit solvent effects of water. This model incorporates the well-known dielectric constant of water (ε = 78.4) to represent the surrounding solvent continuum. This combined DFT/semiempirical methodology has been successfully applied in our previous work on cyclodextrin supramolecular complexes, demonstrating its effectiveness for such systems.^28^ Gaussian 09 served as the computational platform for all theoretical calculations within this study.^29^

## 3 Results and Discussion

### 3.1 Inclusion of SDBS in βCD–NH_2_ Studied by SPR

#### 3.1.1 Kinetic Analysis

**Figure 1:**
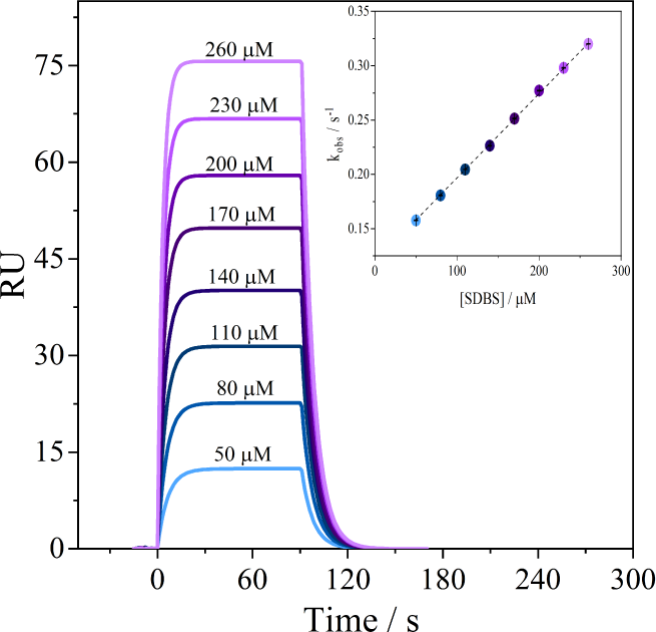
SPR sensorgrams for the formation of complexes between the immobilized βCD–NH_2_ and different concentrations of SDBS (50–260 μM), at pH 7.4 and 298.2 K. Inset: *k*_obs_ vs [SDBS]_*T*_ curve obtained at pH 7.4 and 298.2 K.

The knowledge of the rates at which guest molecules associate with or dissociate from CDs can provide the means to understand the relationship between structure and molecular dynamics,^30^ thus allowing us to describe in detail the complexation mechanism. With this information in mind, we performed time-domain studies of the interaction between SDBS and βCD–NH_2_ using the SPR technique. During the experiment, the absence or presence of SDBS molecules in the solution that flow over the sensor chip with immobilized βCD–NH_2_ altered the resonance angle (θ) at which the SPR phenomenon occurs. The variation of θ over time is the output signal of the equipment, termed a sensorgram, which is typically expressed in resonant units (RU ≅ 0.122 millidegree) per second, as shown in Figure 1. Sensorgrams obtained at different temperatures are presented in Figure S1.

The initial flow of buffer solution over the gold/dextran/CDs surface of the sample and reference cells established a baseline (RU ≅ 0), showing the absence of net intermolecular interactions at this point in the experiment. By injecting solutions with increasing concentrations of SDBS (at *t* = 0), the exponential increase of the RU response indicates a change in the mass adsorbed on the sensor chip caused by the interaction of the surfactant with the immobilized βCD–NH_2_. Because the SPR experiments are conducted in a flow system, some of the surfactant molecules that interact are carried away from the immobilized βCD–NH_2_ during the contact time (0–90 s). This means that this step of the experiment involves a dynamic process of association between free SDBS and βCD–NH_2_ molecules and the dissociation of the thermodynamically stable complexes ([βCD–NH2/SDBS]°) formed. Although both processes occur simultaneously, the association between the molecules predominates, as shown by the increase in the RU.

Based on previous thermodynamic and structural studies that confirm the formation of 1:1 ICs between SDBS and βCD,^17,19,20^ the complexation process can be described by eq 1.
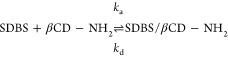
1

Here, *k*_a_ and *k*_d_ are the association and dissociation rate constants. Assuming that the total concentration of SDBS is significantly higher than the total concentration of immobilized βCD–NH_2_, ensuring that the free SDBS concentration remains approximately constant and equal to the total injected concentration throughout the experiment, the dynamics of the complexation process described in eq 1 can be effectively modeled by a pseudo-first-order rate law (eq 2).
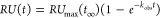
2

Here, *RU*(*t*) represents the RU response at the ascending region of the sensorgram, which is directly proportional to the amount of the [βCD–NH_2_/SDBS]° complex formed over time. *RU*_max_(*t*)_∞_ denotes the maximum capacity for analyte binding at a specific SDBS concentration. It corresponds to the SPR signal when *t* → ∞, signifying the point at which the maximum number of complexes form. *k*_obs_ is the observed rate constant, defined by eq 3, the plot of which is depicted in Figure 1.
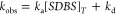
3

After reaching a plateau, the SDBS solution was replaced with the buffer to promote the dissociation of the complexes, resulting in an exponential decrease in the RU values. Because the dissociation takes place mainly in this step, the descending region of the sensorgram is described by a first-order rate law (eq 4).
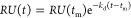
4

Here, *RU*(*t*_m_) is the SPR signal at the beginning of the dissociation step, at *t*_m_. Finally, after some time with only buffer flowing over the sensor chip, the SPR response returned to a baseline, showing that the binding phenomena no longer occur in the system, and the thermodynamically stable complexes were dissociated entirely.

By globally fitting the sensorgrams to eqs 2 and 4, we obtained the *k*_a_ and *k*_d_ values at temperatures ranging from 285.2–301.2 K, listed in Table 1.

**Table 1:** *k*_a_ and *k*_d_ Values of [βCD–NH_2_/SDBS]°, at 285.2–301.2 K and pH 7.4

*T*	*k*_a_	*k*_d_
K	10^2^ M^–1^ s^–1^	10^–2^ s^–1^
285.2	6.00 ± 0.05	7.10 ± 0.03
289.2	7.80 ± 0.06	8.75 ± 0.03
293.2	8.60 ± 0.07	10.8 ± 0.1
297.2	8.2 ± 0.1	13.0 ± 0.2
298.2	7.80 ± 0.07	13.8 ± 0.3
301.2	6.50 ± 0.05	16.3 ± 0.3

**Figure 2:**
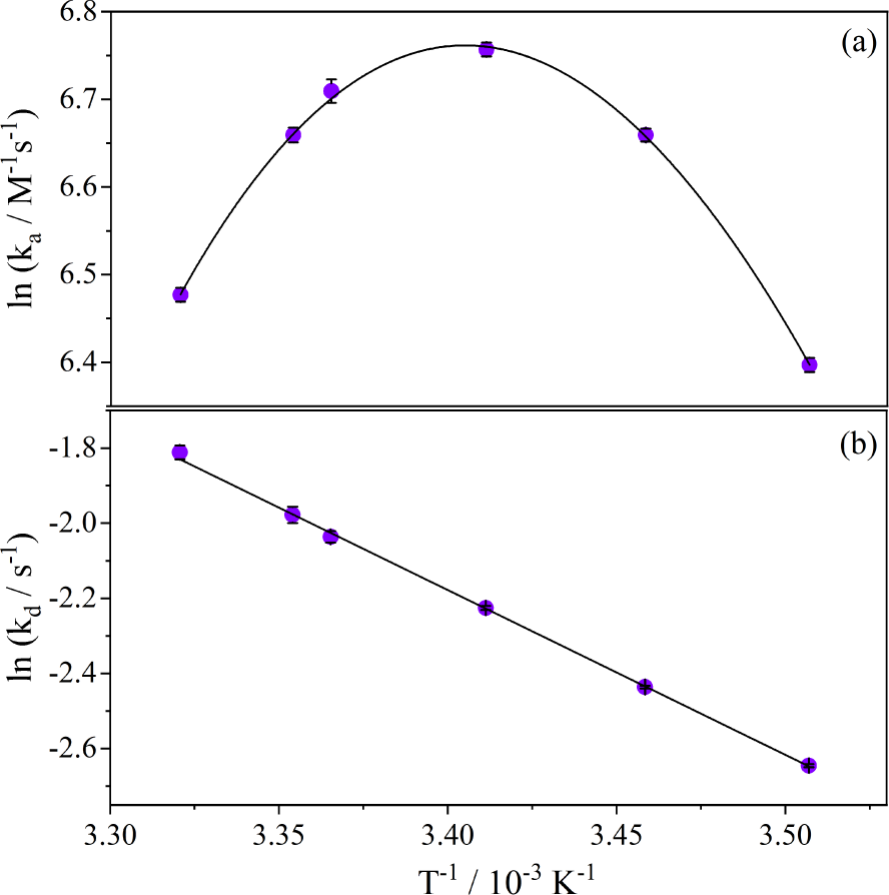
Arrhenius plots of (a) ln *k*_a_ and (b) ln *k*_d_ associated with the interaction between SDBS and βCD–NH_2_ as functions of reciprocal temperatures.

The *k*_a_ values represent the number of complexes formed per second. For the [βCD–NH_2_/SDBS]° system, these values were 10^2^ M^–1^ s^–1^, displaying a nonlinear behavior with increasing temperature. In contrast, the *k*_d_ values, indicating the fraction of [βCD–NH_2_/SDBS]° that dissociates per second, were 10^–2^ s^–1^ and increased with rising temperature. The rate constants at which the host and guest molecules associate and the resulting complex dissociates depend directly on the various molecular processes occurring in the system. These processes include hydrophobic, polar, or steric interactions, hydrogen bonding, torsion of the CD ring, and the release of solvation water molecules.^31^ To elucidate these processes’ role in modulating the inclusion phenomenon’s kinetics, the energetic parameters governing the βCD–NH_2_/SDBS interaction were quantified. Initially, an analysis of the dependence of the kinetic constants on the temperature was conducted through Arrhenius plots, as shown in Figure 2.

For the association of free SDBS molecules and free immobilized βCD–NH_2_, ln *k*_a_ values showed a polynomial behavior at the studied reciprocal temperature range, as described by eq 5, where *a*, *b*, *c*, and *d* are constants obtained by fitting the curve shown in Figure 2a.
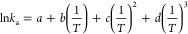
5

The deviation from linearity in the Arrhenius plot typically indicates complexity in the binding mechanism. This complexity suggests that the activation energy required for the system to reach the transition state differs at each temperature, indicating a multistep process.^32^ According to the transition state theory, the activation energy () for the formation of the activated IC ([βCD–NH_2_/SDBS]^‡^) from the association of the free molecules can be obtained through the first derivative of ln *k*_a_ with respect to , resulting in eq 6.
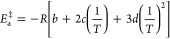
6

Meanwhile, ln*k*_d_ varied linearly with the reciprocal temperature showing that the dissociation of the [βCD–NH_2_/SDBS]° into [βCD–NH_2_/SDBS]^‡^ occurs in a single-step and with activation energy () calculated from the slope of the ln *k*_d_ vs  curve (Figure 2). From the values of , where *x* = a (association) or *x* = d (dissociation), the changes in the enthalpy (Δ*H*_*x*_^‡^), free Gibbs energy (Δ*G*_*x*_^‡^), and entropy () of activation (assuming the standard concentration of 1 mol L^–1^) were calculated through eqs 7, 8 and 9, respectively.
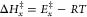
7
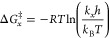
8
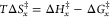
9

**Figure 3:**
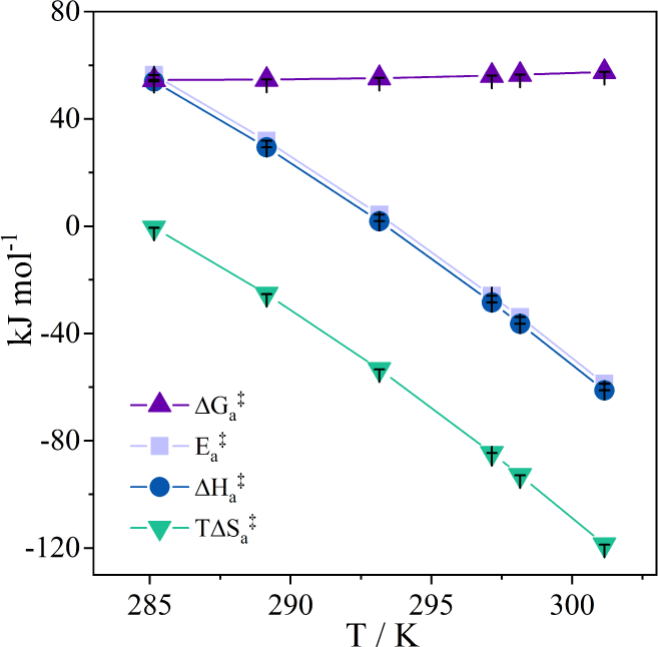
Temperature dependence of the energetic parameters for the formation of [βCD–NH_2_/SDBS]^‡^ by association of free SDBS and free immobilized βCD–NH_2_ at pH 7.4.

Here, *h* and *k*_B_ are the Planck and Boltzmann constants, respectively. Figure 3 shows the energetic parameters for the formation of [βCD–NH_2_/SDBS]^‡^ from the association of free molecules as a function of the temperature.

At the investigated temperature range, the  values were positive and remained relatively constant. This parameter reflects the free energy that separates the transition state from the free interacting molecules and results from the potential energy ( and ) and configurational/conformational entropy () changes in the system during the formation of [βCD–NH_2_/SDBS]^‡^.

At each temperature, each molecular process contributed distinctively to the resulting , , and  values. From 285.2 to 293.2 K, the energy absorbed by the system to desolvate the SDBS tail^17^ exceeds the energy released in other processes, such as the desolvation of the βCD–NH_2_ cavity,^33^ and the establishment of intermolecular interactions, primarily van der Waals forces, between the molecules. However, in this temperature range, the entropy gained by the water molecules leaving the SDBS solvation layer is insufficient to yield a positive , resulting in negative  promoted by reducing each interacting molecule’s translational degree of freedom. As the temperature rises, the enthalpic and entropic contributions from SDBS desolvation diminish, leading to even lower  values and negative  and  values in the range of 297.2 to 301.2 K. The reduced contribution of the SDBS desolvation process to the , , and  values at higher temperatures is attributed to the increase in average translational kinetic energy of the water molecules. This increase causes the water molecules in the SDBS solvation layer to become less structured and more similar to those in the bulk, thereby reducing the energy absorbed and the entropy gained from desolvating the surfactant.

**Figure 4:**
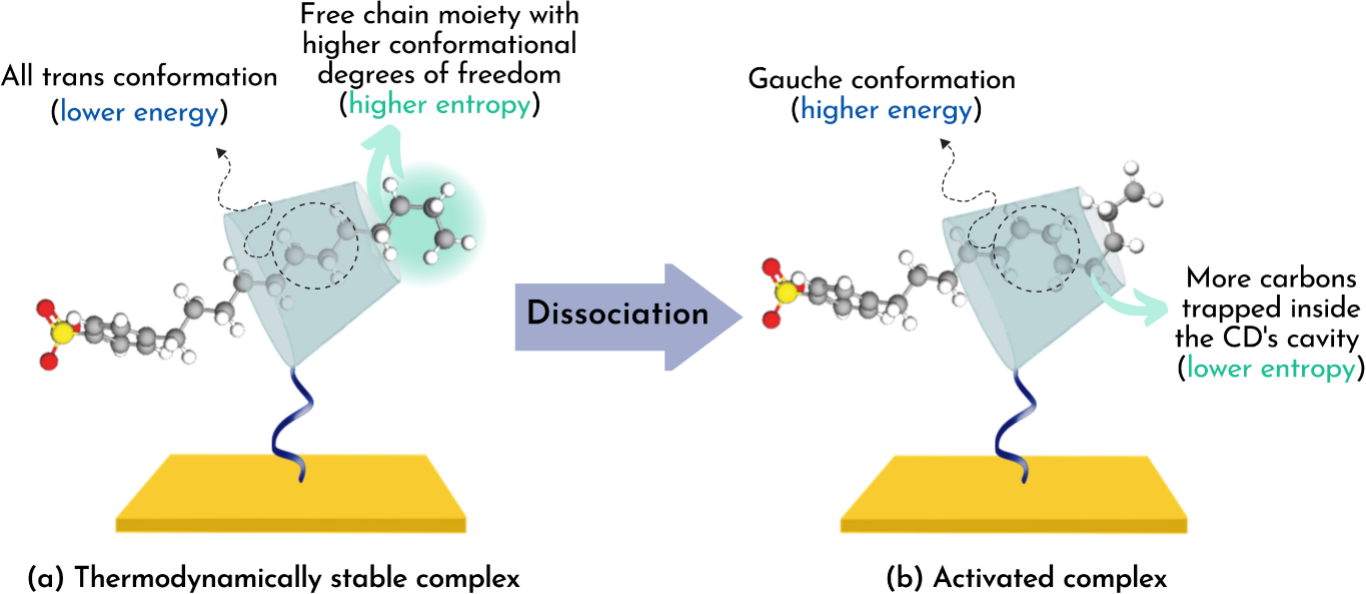
Representation of the dissociation of the (a) thermodynamically stable complex [βCD–NH_2_/SDBS]° into the (b) activated complex [βCD–NH_2_/SDBS]^‡^.

When [βCD–NH_2_/SDBS]^‡^ is formed from the dissociation of [βCD–NH_2_/SDBS]°, on the other hand, the results showed that all of the energetic parameters related to this process did not depend on the temperature (Table S1). This behavior can be attributed to the fact that, at this step, most of the SDBS desolvation had already taken place, which was the leading cause for the association step to be temperature-dependent. Instead, the value of  was 36.783 ± 0.001 kJ mol^–1^, while the average values of  and  were 34.3 ± 0.1 kJ mol^–1^ and −43.0 ± 0.9 kJ mol^–1^, respectively. These , , and  values obtained indicate that the dissociation of [βCD–NH_2_/SDBS]° into [βCD–NH_2_/SDBS]^‡^ occurs with a restructuration of the ICs. Based on estimations by 2D ROESY measurement performed by Ogoshi et al., in the thermodynamically stable complex the SDBS molecule is partially included by βCD–NH_2_, with the macrocyclic molecule located at the middle of the alkyl chain,^20^ as shown in Figure 4a. In this state, the fraction included by βCD–NH_2_, approximately four carbon atoms, is in an all-trans conformation (lower energy). However, based on geometric considerations, βCD can accommodate eight carbon atoms forming gauche conformations, which causes the chain to form kinks.^34^ Because *gauche*/*gauche* bonds in a hydrocarbon chain are more energetic than those in an all-*trans* conformation, inserting more segments of the alkyl chain in the βCD–NH_2_ cavity increases the potential energy barrier of the system. Consequently, the alkyl chain becomes spatially restricted, thus decreasing its entropy. Based on this information, our results could indicate that the energy absorption and entropy loss required by [βCD–NH_2_/SDBS]° to dissociate into [βCD–NH_2_/SDBS]^‡^ results from the torsion of the alkyl chain to fit more carbons in the cavity (Figure 4b). This could mean that in the activated state the included SDBS chain retains part of its random coiled conformation assumed when free in solution, which becomes extended when the [βCD–NH_2_/SDBS]° forms.

#### 3.1.2 Thermodynamic Analysis

While the kinetic analysis allows uncovering of the path the free molecules have to go through to form the thermodynamically stable complex, a thermodynamic study informs us about the stability and driving forces of the complex formed. Because there is a relationship between kinetics and thermodynamics, we can investigate the latter through the former. This relation is possible because the energetic parameters obtained for forming [βCD–NH_2_/SDBS]^‡^, either by the association or dissociation steps, contain information about the uncomplexed and complexed states of the molecules, respectively. Therefore, once the activated properties are known, the thermodynamic parameters for the inclusion process can be calculated as follows,^35^
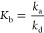
10
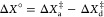
11

where the function *X* can be *G*, *H*, or *S*. By using this method, we calculated the thermodynamic parameters for the [βCD–NH_2_/SDBS]° formation at the temperature range of 285.2–301.2 K, which are listed in Table 2.

**Table 2:** Thermodynamic Parameters for the Formation of [βCD–NH_2_/SDBS]°, at pH 7.4 and Different Temperatures

*T* (K)	*K*_b_ (10^3^ L mol^–1^)	Δ*G*° (kJ mol^–1^)	Δ*H*° (kJ mol^–1^)	*T*Δ*S*° (kJ mol^–1^)
285.2	8.45 ± 0.08	–21.4374 ± 0.0001	19.60 ± 0.02	41.04 ± 0.02
289.2	8.91 ± 0.07	–21.8665 ± 0.0001	–4.91 ± 0.02	16.95 ± 0.02
293.2	7.96 ± 0.08	–21.8939 ± 0.0001	–32.44 ± 0.02	–10.55 ± 0.02
297.2	6.31± 0.1	–21.6169 ± 0.0002	–62.71 ± 0.02	–41.09 ± 0.02
298.2	5.7 ± 0.1	–21.4177 ± 0.0002	–70.68 ± 0.02	–49.26 ± 0.02
301.2	3.99 ± 0.08	–20.7598 ± 0.0002	–95.47 ± 0.02	–74.71 ± 0.02

The *K*_b_ values obtained by SPR were in the order of 10^3^ L mol^–1^, which were similar to the result obtained by Liu et al. for the interaction between SDBS and βCD at 298.2 K, using isothermal titration calorimetry.^19^ Additionally, our results demonstrated that the stability of the ICs formed between SDBS and βCD–NH_2_ depended little on the temperature increase, as the average Δ*G*° value in the 285.2–301.2 K range was −21.5 ± 0.4 kJ mol^–1^. This behavior results from a balance between the enthalpic and entropic contributions as the temperature increases, which maintains the system’s stability. This phenomenon, known as enthalpy–entropy compensation (EEC), is commonly observed in systems where the solvent reorganization is a determinant factor, such as the formation of CD-based ICs.^36,37^ This hypothesis was confirmed by the EEC plot shown in Figure S2. The linear regression of the Δ*H*° vs *T*Δ*S*° curve gave a slope close to the unit (0.99), which is indicative of the invariability of Δ*G*°.^36^

On the other hand, the Δ*H*°and *T*Δ*S*° depended highly on the temperature as the inclusion process goes from entropy-driven at low temperature (285.2 K) to enthalpy-driven as the temperature increases (≥293.2 K). As discussed in the kinetics section, the temperature increase mainly affects the surfactant’s desolvation, decreasing its contribution to the resulting Δ*H*°and *T*Δ*S*° values. Consequently, the desolvation of the βCD–NH_2_ cavity, the tail–cavity interaction, and the transition of the SDBS chain from a random coil to an all-trans conformation become the driving processes for the formation of [βCD–NH_2_/SDBS]°.

### 3.2 Effect of C_4_mimCl on the βCD–NH_2_/SDBS Inclusion Process

Both kinetic and thermodynamic analyses of the complexation between SDBS and βCD–NH_2_ in buffer showed how the solvation water molecules play a crucial role in the inclusion process. Besides the temperature dependence, another way to investigate how the desolvation of the surfactant affects the inclusion is by adding a cosolute and checking how the system behaves in its presence. With this purpose, we studied how the presence of IL C_4_mimCl affects the inclusion of SDBS in βCD–NH_2_ from kinetic and thermodynamic perspectives. The sensorgrams obtained are shown in Figures S3–S6 and the resulting *k*_a_ and *k*_d_values are listed in Table S2.

**Figure 5:**
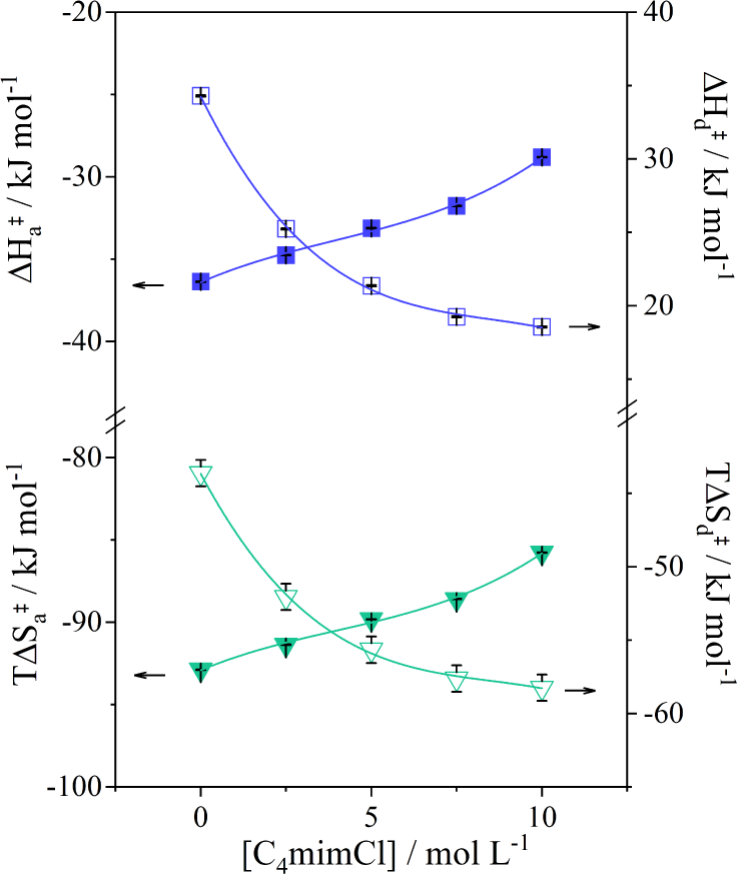
Activated enthalpy and entropy changes as a function of C_4_mimCl concentration for the formation of the activated complex from the association between SDBS and βCD–NH_2_ (solid square and solid inverted triangle) or the dissociation of the thermodynamically stable complex (open square and open inverted traingle) at 298.2 K and pH 7.4.

The formation of [βCD–NH_2_/SDBS]^‡^ in the presence of C_4_mimCl depended on the temperature the same way as in the absence of the IL, in which the association (Figure S7a) and dissociation (Figure S7b) routes were a multistep and a single-step process, respectively. At each temperature, the increase of the C_4_mimCl concentration had almost no effect on the free energy barriers. For instance, at 298.2 K, and  at the [C_4_mimCl] range of 0 to 10 mM. These results indicate that [βCD–NH_2_/SDBS]^‡^ is formed through similar processes to when there is no cosolute present, but with different magnitudes of  and  as the C_4_mimCl concentration increases, as shown by Figure 5. Data obtained at other temperatures are listed in Tables S3–S6.

At 298.2 K and with increasing concentration of C_4_mimCl, the formation of [βCD–NH_2_/SDBS]^‡^ from the association between free SDBS and free immobilized βCD–NH_2_ occurred with the increase of  and . Although the C_4_mim^+^ cation can form IC with βCD, the stability of the complex formed () at 298.0 K^38^ is not high enough for the IL to compete with SDBS for the cavity. Therefore, the increase of  and  can be attributed to changes in the desolvation of SDBS.

The C_4_mim^+^ cation can perform attractive electrostatic interaction and π–π stacking with the headgroup and benzyl group of SDBS, respectively. This interaction could neutralize the surfactant’s charge, increasing its hydrophobicity. As a result, the enthalpic and entropic content of the water molecules on the solvation layer of SDBS increases, causing the desolvation process to occur with a more significant increase in the system’s enthalpy and entropy. This change in the solvation layer of the surfactant probably also affects its conformation when free in solution by favoring a more random coiled conformer. As discussed previously, this conformation is partially retained when [βCD–NH_2_/SDBS]^‡^ is formed.

**Figure 6:**
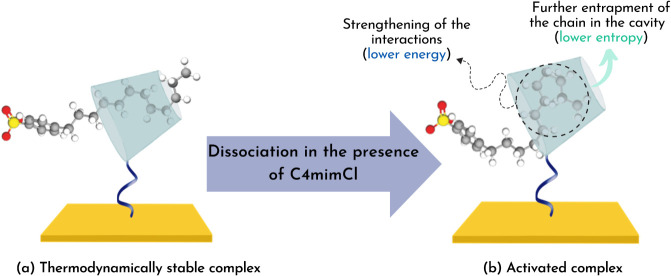
Representation of the dissociation of the (a) thermodynamically stable complex [βCD–NH_2_/SDBS]° into the (b) activated complex [βCD–NH_2_/SDBS]^‡^.

The results from the dissociation step corroborate this hypothesis. The increase in the IL concentration caused the decrease of the  and  values. The positive  and negative  values, in the absence of C_4_mimCl, were attributed to the insertion of more carbon atoms in the βCD–NH_2_ cavity when [βCD–NH_2_/SDBS]° dissociates into [βCD–NH_2_/SDBS]^‡^. Therefore, the decrease of  and , as the concentration of C_4_mimCl increased, was probably caused by the further insertion of the SDBS chain in the cavity (Figure 6). When this occurs, the surfactant loses more degrees of freedom as kinks are formed. Moreover, the intermolecular (tail–cavity) interactions are enhanced, counterbalancing the potential energy barrier imposed by the gauche conformation that the chain assumes in the cavity.

From the activated parameters, the thermodynamics of the [βCD–NH_2_/SDBS]° formation were calculated as described by eqs 10 and 11, and are listed in Table 3. Data obtained at other temperatures are listed in Tables S7–S10.

**Table 3:** Thermodynamic Parameters for the Formation of [βCD–NH_2_/SDBS]° in the Presence of Varying Concentrations of C_4_mimCl at 298.2 and pH 7.4

[C_4_mimCl] (mM)	*K*_b_ (10^3^ L mol^–1^)	Δ*G*° (kJ mol^–1^)	Δ*H*° (kJ mol^–1^)	*T*Δ*S*° (kJ mol^–1^)
0	5.7 ± 0.1	–21.4177 ± 0.0002	–70.67 ± 0.05	–49.25 ± 0.05
2.5	4.20 ± 0.08	–20.6798 ± 0.0002	–59.99 ± 0.05	–39.31 ± 0.05
5.0	3.61 ± 0.06	–20.3044 ± 0.0002	–54.48 ± 0.05	–34.18 ± 0.05
7.5	3.25 ± 0.05	–20.0479 ± 0.0001	–51.01 ± 0.05	–30.96 ± 0.05
10	2.98 ± 0.05	–19.8300 ± 0.0001	–47.37 ± 0.05	–27.54 ± 0.05

The increase of C_4_mimCl concentration up to 10 mM affected the stability of [βCD–NH_2_/SDBS]°, as shown by the reduction in 47% and 7% of *K*_b_ and Δ*G*°, respectively. This decrease in complex stability results from Δ*H*° increasing more rapidly than *T*Δ*S*°as the IL concentration grew.

At 298.2 K, the main driving forces for the formation of [βCD–NH_2_/SDBS]° are the desolvation of the βCD–NH_2_ (Δ*H <* 0 and *T*Δ*S <* 0), the intermolecular interactions performed by the pair (Δ*H <* 0 and *T*Δ*S <* 0), and the conformational changes suffered by the surfactant (Δ*H <* 0 and *T*Δ*S <* 0), which overcome the desolvation of the SDBS hydrophobic tail (Δ*H >* 0 and *T*Δ*S >* 0). As discussed in the kinetics analysis, the increase in C_4_mimCl concentration mainly affects the release of water molecules from the SDBS solvation layer and the conformation the surfactant assumes in the solution. Thus, the increase of Δ*H*° and *T*Δ*S*° values is probably caused by (a) more energy required to desolvate the SDBS tail and, consequently, more entropy gained by the water molecules when released to the bulk and (b) less energy released and entropy lost from the *gauche* to *trans* conformational transition due to the SDBS tail retaining part of its in-solution conformation inside the βCD–NH_2_ cavity.

### 3.3 Theoretical Studies

**Figure 7:**
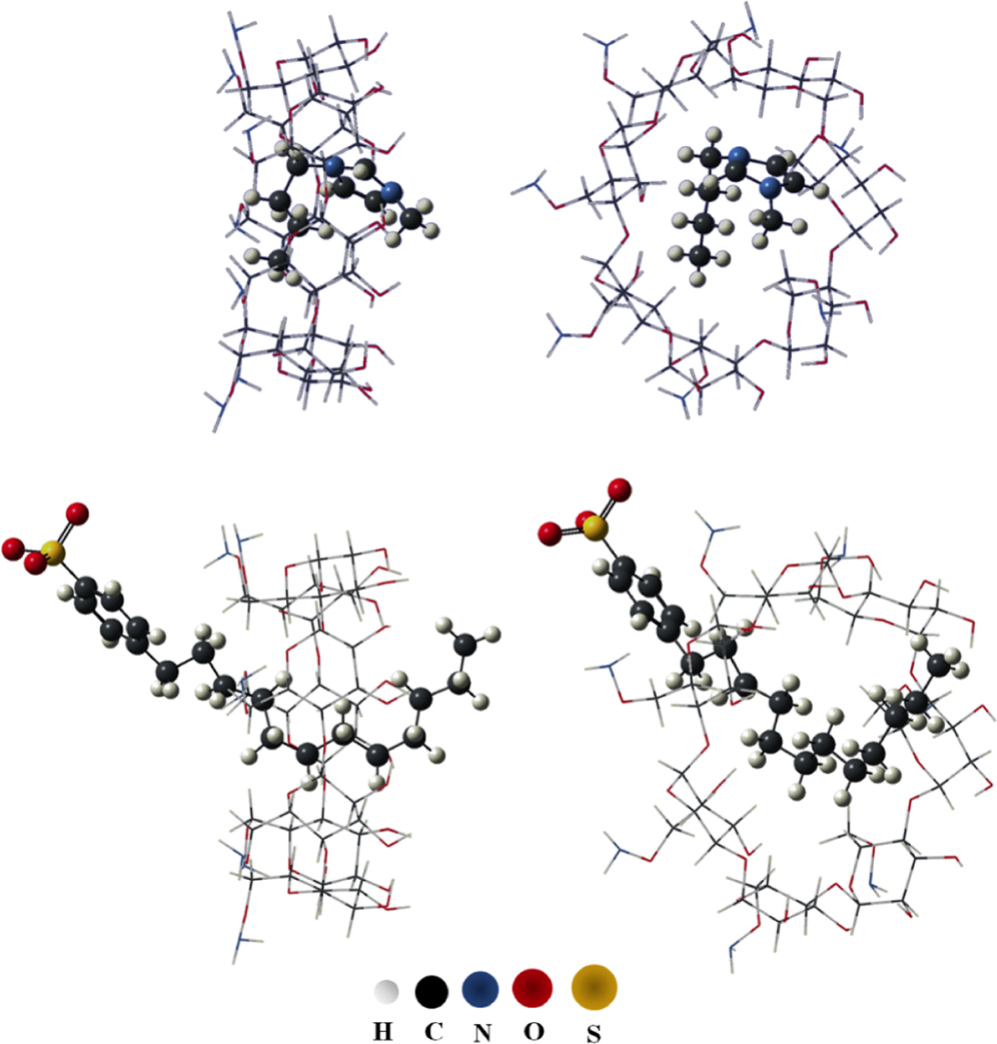
PM3 fully optimized geometry of the βCD–NH_2_/C_4_mim^+^ and βCD–NH_2_/SDBS in two views.

The thermodynamic properties (Table 4), including enthalpy (Δ*H*) and Gibbs free energy (Δ*G*) changes, of βCD–NH_2_/C_4_mim^+^ and βCD–NH_2_/SDBS complexes were determined using B97*D*/6-31G (d,p)//PM3 computational methods. The optimized molecular geometries for these complexes are listed in Figure 7.

**Table 4:** Δ*H* and Δ*G* Obtained from B97*D*/6-31(d,p)//PM3 Calculations in Aqueous Solution (SMD Method) for the ICs Formed by C_4_mim^+^ or SDBS and βCD–NH_2_

Complex	Δ*H* kJ mol^–1^	ΔG kJ mol^–1^
βCD–NH_2_/C_4_mim^+^	–47.7	–5.4
βCD–NH_2_/SDBS	–81.6	–25.5

The results confirmed the spontaneous formation of ICs between βCD–NH_2_ and SDBS, thermodynamically favored toward an equilibrium state. This suggests a partial intercalation mechanism in which the SDBS molecule inserts itself partially into the βCD–NH_2_ cavity. The intrinsic conformational flexibility of the SDBS alkyl tail facilitates this intercalation process. The contorted nature of the tail allows for deeper insertion of carbon atoms into the βCD–NH_2_ cavity, promoting the establishment of weak intermolecular interactions. These interactions, primarily London dispersion forces, are majorly responsible for the enhanced stability of the encapsulated SDBS within the macrocyclic cavity. These findings align congruently with the observations obtained through SPR experiments.

In contrast, although the C_4_mim^+^ cation can also form an IL with βCD–NH_2_, the stability of this complex is not sufficiently high for the IL to compete with SDBS in the cavity. Therefore, due to the formation of more stable ICs, SDBS is preferentially incorporated by βCD–NH_2_.

## 4 Conclusion

This paper investigated the kinetics and thermodynamics of IC formation based on the interaction between SDBS and βCD–NH_2_ using the SPR technique and theoretical studies. The obtention of the rate constants at different temperatures showed that the molecules formed an activated complex before the thermodynamically stable complex. Starting from the association of free SDBS and immobilized βCD–NH_2_, the formation of [βCD–NH_2_/SDBS]^‡^ was shown to be a multistep process in which, , and  decreased as the temperature increased. Contrary to the association route, the dissociation of [βCD–NH_2_/SDBS]° into [βCD–NH_2_/SDBS]^‡^ was a single-step process accompanied by invariable positive  and , and negative  values throughout the temperature range studied. From these results, a mechanism for the dissociation was proposed in which the tail moiety encapsulated by βCD–NH_2_ goes from an all-*trans* conformation (in the thermodynamically stable complex) to a more coiled conformation (in the activated complex). Moreover, the formation of kinks in the SDBS tail, when included by βCD–NH_2_, allows more carbon atoms inside the cavity of the activated complex. This event was even more pronounced when ICs were formed in the presence of C_4_mimCl. While from these kinetic data we could provide a whole movie of the inclusion, from both energetic and structural perspectives, the thermodynamic analysis revealed a shift from an entropy-driven to an enthalpy-driven complexation process as temperature increased, with increasing concentrations of C_4_mimCl decreasing the stability of the complex. These findings contribute to a further understanding of the dynamics of supramolecular systems and the different structures the complexes assume throughout the complexation process.

## Supporting Information Available

Sensorgrams for βCD–NH_2_ interacting with different concentrations of SDBS at various temperatures; energetic parameters for the formation of the activated complex from the dissociation of the thermodynamically stable complex; enthalpy–entropy compensation plot; sensorgrams for βCD–NH_2_ interacting with different concentrations of SDBS in the presence of 2.5–10 mM of C_4_mimCl at various temperatures; *k*_a_ and *k*_d_ values of [βCD–NH_2_/SDBS]° in the presence of different C_4_mimCl at various temperatures; plots of ln *k*_a_ and ln *k*_d_ versus 1/*T* for βCD–NH_2_/SDBS-activated complex formation in the presence of the C_4_mimCl; energetic parameters for the formation of the activated complex in the presence of 2.5–10 mM of C_4_mimCl at various temperatures; thermodynamic parameters for the formation of [βCD–NH_2_/SDBS]° in the presence of 2.5–10 mM of C_4_mimCl at various temperatures (PDF)
