# Supplementary material for: Exploring the Recognition Mechanism of Surfactant–Cyclodextrin Complex Formation: Insights from SPR Studies on Temperature and Ionic Liquid Influence
Source: J Phys Chem B. 2024 Sep 20;128(39):9604–12. doi: 10.1021/acs.jpcb.4c04516 (PMC11457137; doi:10.1021/acs.jpcb.4c04516)
Supplement: Supplementary file 1 — jp4c04516_si_001.pdf [file jp4c04516_si_001.pdf]

Supporting Information

Exploring the Recognition Mechanism of Surfactant-Cyclodextrin Complex Formation: Insights from SPR Studies on Temperature and Ionic Liquid Influence

Isabela Araujo Marques[a]
Hauster Maximiler C. de Paula[a]
Camilla Fonseca Silva[b]
Clebio Soares Nascimento Jr.[b]
Yara Luiza Coelho[c]
Ana Clarissa dos Santos Pires[d]
Luis Henrique Mendes da Silva[a]*

[a]Advanced Thermokinetics of Molecular Systems (ATOM) Group, Chemistry Department, Federal University of Viçosa, PH Rolfs Avenue, 36570-000 Viçosa-MG, Brazil. [b]Theoretical and Computational Chemistry (LQTC) Laboratory, Department of Natural Sciences (DCNAT), Federal University of São João Del Rei, Dom Bosco Campus, 36301-160 São João Del Rei-MG, Brazil. [c]Colloid Chemistry Group, Chemistry Institute, Federal University of Alfenas, 700 Gabriel Monteiro da Silva Street, 37130-000 Alfenas-MG, Brazil. [d]Applied Molecular Thermodynamic (THERMA), Food Technology Department, Federal University of Viçosa, PH Rolfs Avenue, 36570-000 Viçosa-MG, Brazil.

* Corresponding author: luhen@ufv.br; Phone: +55 31 38993052.

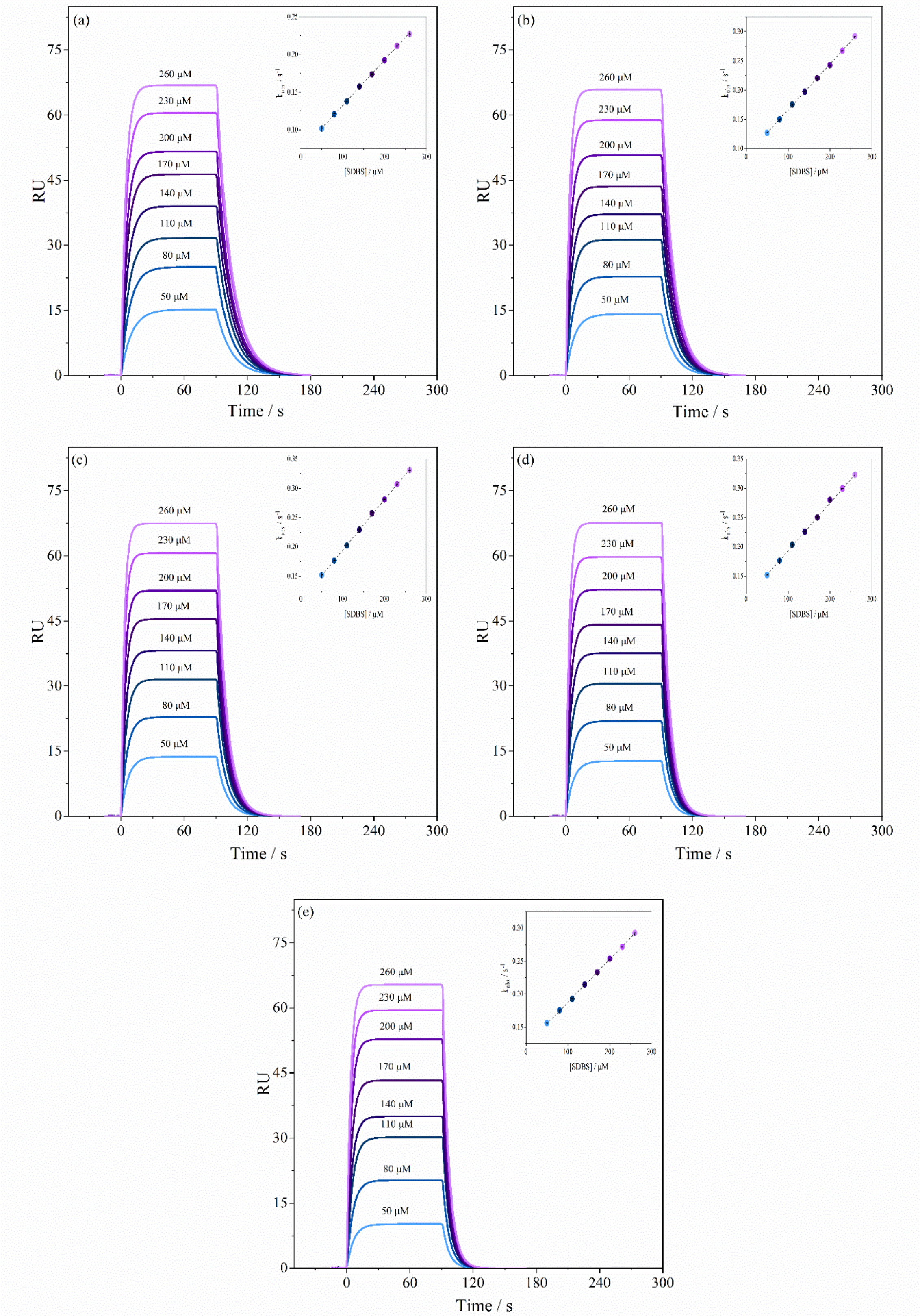

Fig. S1. SPR sensorgrams for the formation of complexes between the immobilized βCD−NH_2 and different concentrations of SDBS (50−260 μM), at pH 7.4 and (a) 285.2 K, (b) 289.2 K, (c) 293.2 K, (d) 297.2 K, and (e) 301.2 K. Inset: k_{obs} vs. $[SDBS]_T$ curve obtained at pH 7.4 and the respective temperatures.

Table S1. Energetic parameters for the formation of the activated complex [βCD–NH_2/SDBS]‡ from the dissociation of the thermodynamically stable complex [βCD–NH_2/SDBS]°, at pH 7.4 and different temperatures.

**T**	$\mathbf{E}_d^‡$	$\mathbf{\Delta H}_d^‡$	$\mathbf{\Delta G}_d^‡$	$\mathbf{T\Delta S}_d^‡$
K	kJ mol^{-1}			
285.2		34.032 ± 0.001	76.01922 ± 0.00004	-41.987 ± 0.001
289.2		33.999 ± 0.001	76.61645 ± 0.00003	-42.617 ± 0.001
293.2	36.404 ± 0.001	33.966 ± 0.001	77.19650 ± 0.00005	-43.231 ± 0.001
297.2		33.933 ± 0.001	77.8250 ± 0.0001	-43.892 ± 0.001
298.2		33.924 ± 0.001	77.9471 ± 0.0002	-44.023 ± 0.001
301.2		33.899 ± 0.001	78.3394 ± 0.0002	-44.440 ± 0.001

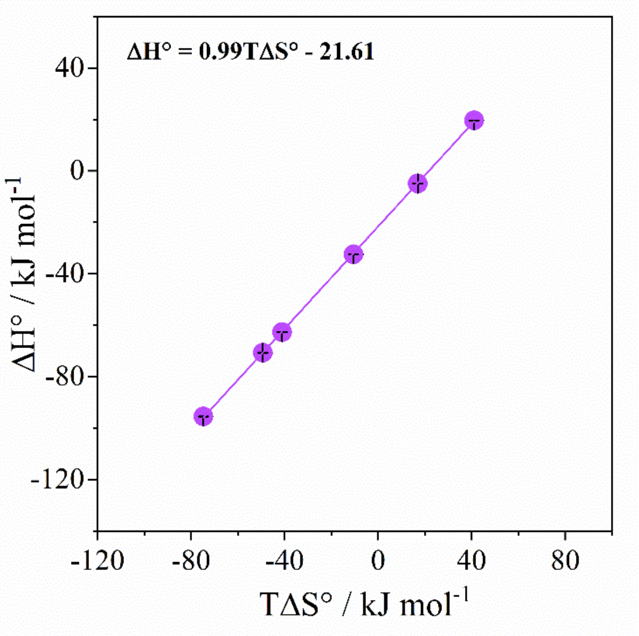

Fig. S2 Enthalpy-entropy compensation plot for the formation of the inclusion complex between SDBS and βCD–NH_2 at pH 7.4.

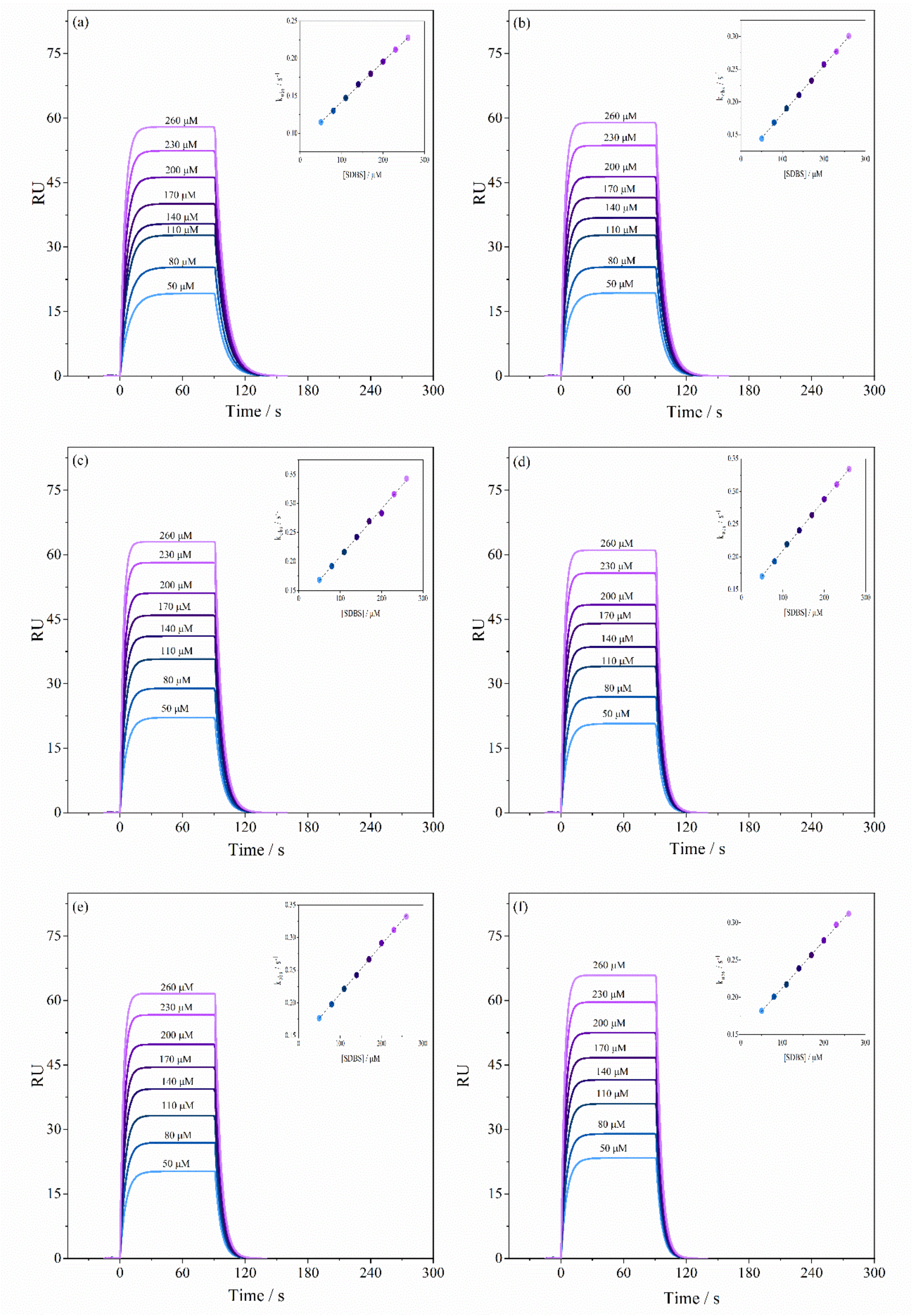

Fig. S3. SPR sensorgrams for the formation of complexes between the immobilized βCD−NH_2 and different concentrations of SDBS (50−260 μM) in 2.5 mM of C_4mimCl, at pH 7.4 and (a) 285.2 K, (b) 289.2 K, (c) 293.2 K, (d) 297.2 K, and (e) 301.2 K. Inset: k_{obs} vs. $[SDBS]_T$ curve obtained at pH 7.4 and the respective temperatures.

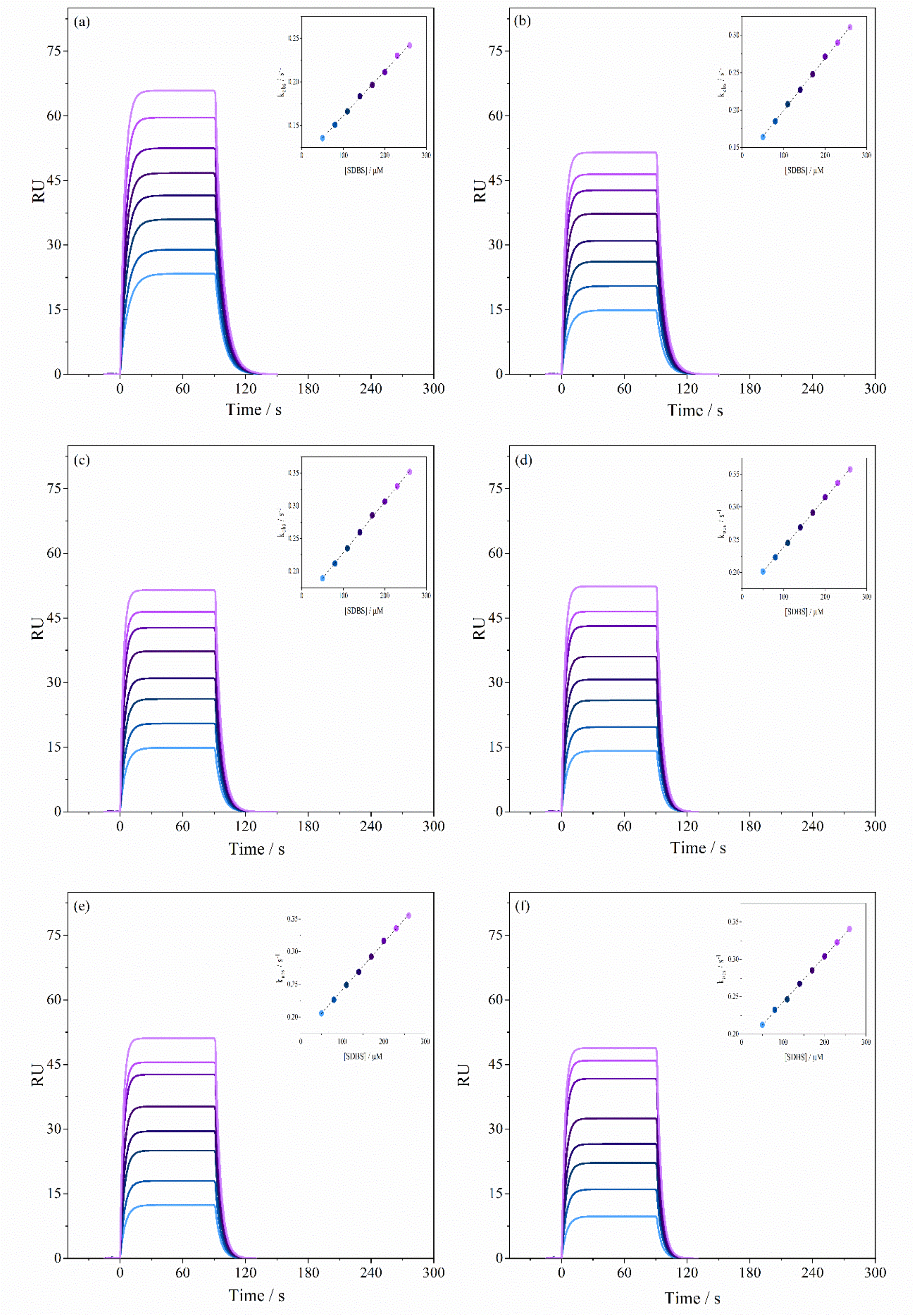

Fig. S4. SPR sensorgrams for the formation of complexes between the immobilized βCD−NH_2 and different concentrations of SDBS (50−260 μM) in 5.0 mM of C_4mimCl, at pH 7.4 and (a) 285.2 K, (b) 289.2 K, (c) 293.2 K, (d) 297.2 K, and (e) 301.2 K. Inset: k_{obs} vs. $[SDBS]_T$ curve obtained at pH 7.4 and the respective temperatures.

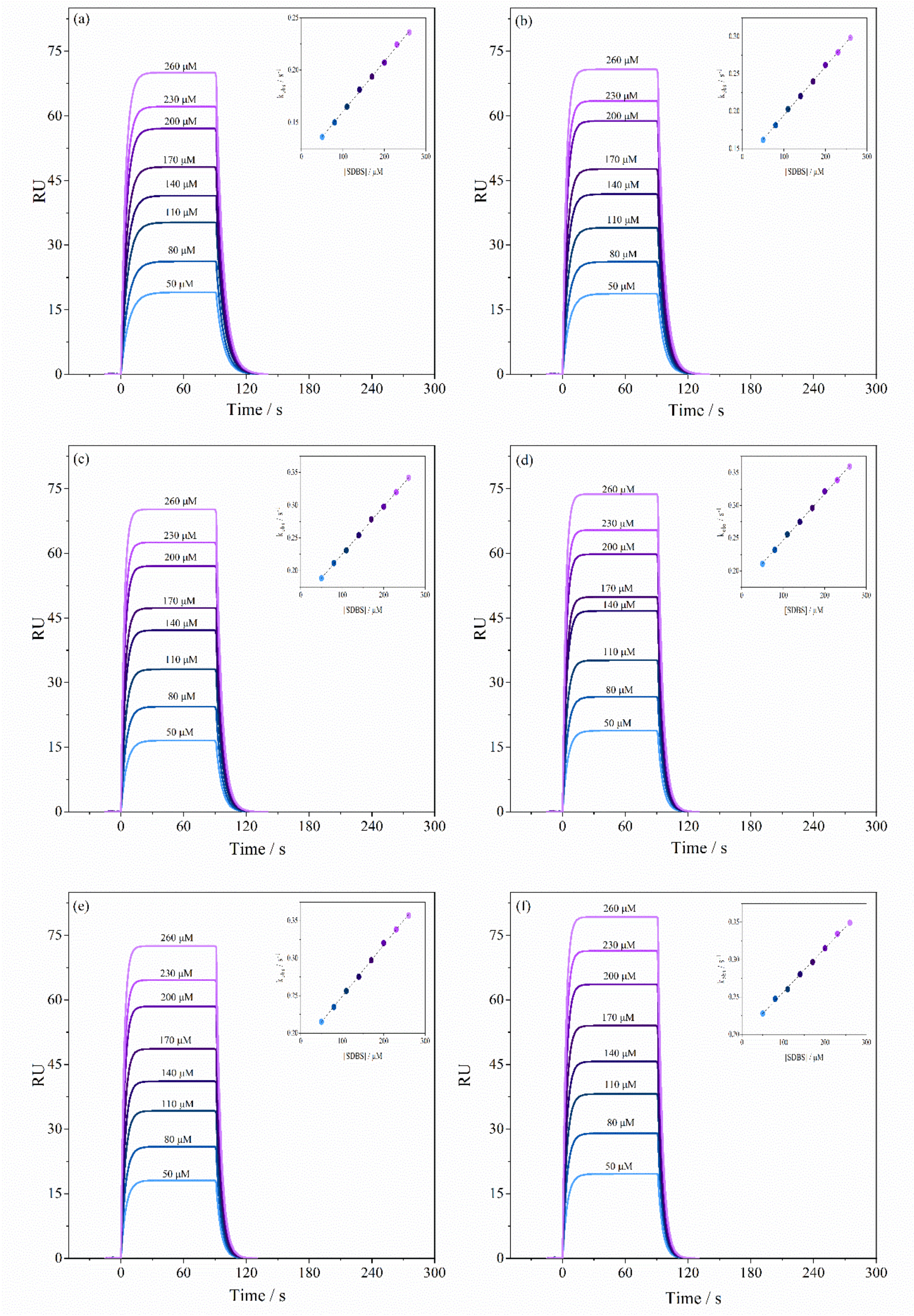

Fig. S5. SPR sensorgrams for the formation of complexes between the immobilized $\beta CD-NH_2$ and different concentrations of SDBS (50−260 μM) in 7.5 mM of C_4mimCl, at pH 7.4 and (a) 285.2 K, (b) 289.2 K, (c) 293.2 K, (d) 297.2 K, and (e) 301.2 K. Inset: k_{obs} vs. $[SDBS]_T$ curve obtained at pH 7.4 and the respective temperatures.

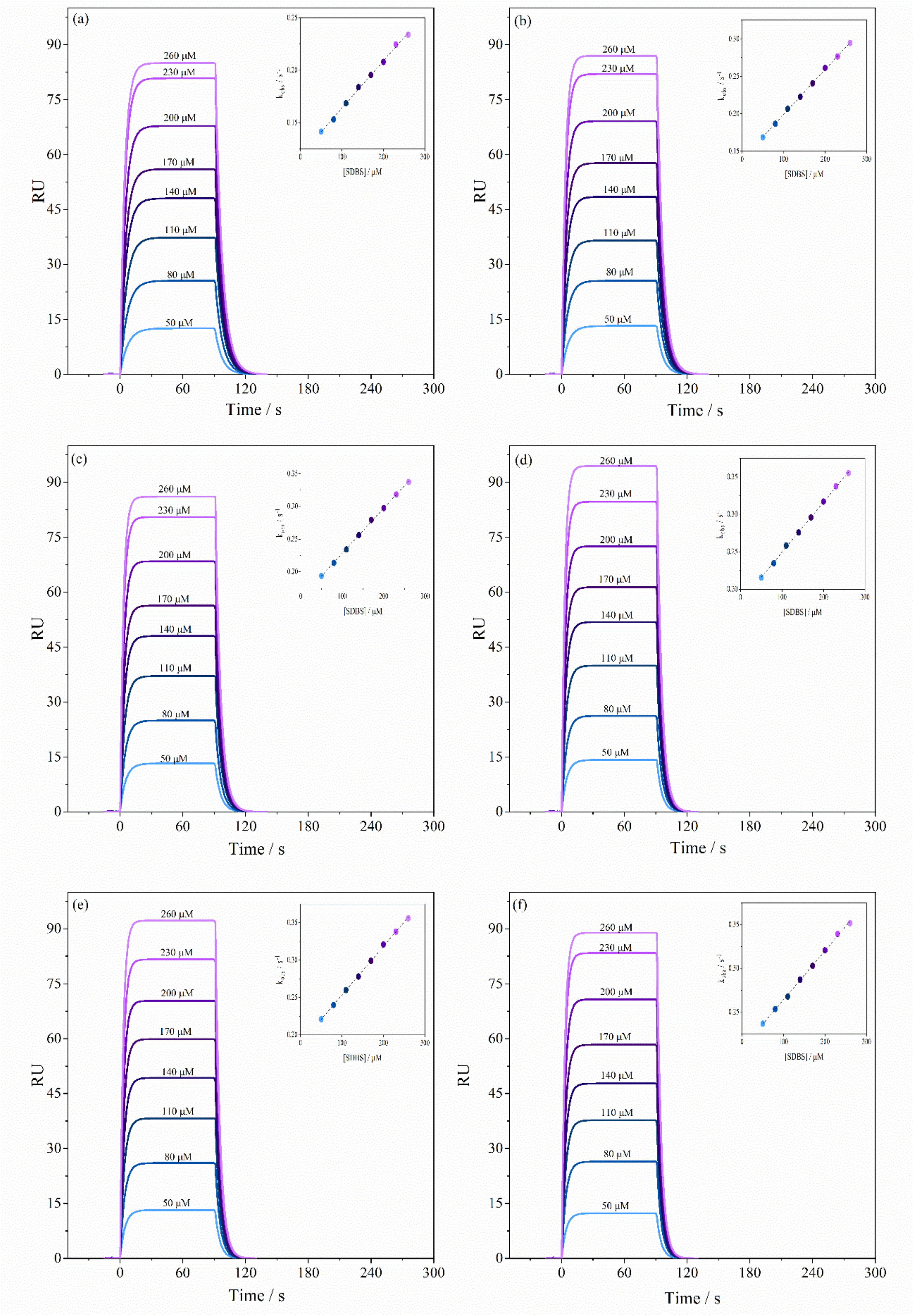

Fig. S6. SPR sensorgrams for the formation of complexes between the immobilized βCD−NH_2 and different concentrations of SDBS (50−260 μM) in 10 mM of C_4mimCl, at pH 7.4 and (a) 285.2 K, (b) 289.2 K, (c) 293.2 K, (d) 297.2 K, and (e) 301.2 K. Inset: k_{obs} vs. $[SDBS]_T$ curve obtained at pH 7.4 and the respective temperatures.

Table S2. k_a and k_d values of [βCD–NH_2/SDBS]° in the presence of different C_4mimCl concentrations, at 285.2–301.2 K and pH 7.4.

**T**	**[C_4mimCl]**							
	2.5 mM		5.0 mM		7.5 mM		10 mM	
	$\mathbf{k_a}$	$\mathbf{k_d}$	$\mathbf{k_a}$	$\mathbf{k_d}$	$\mathbf{k_a}$	$\mathbf{k_d}$	$\mathbf{k_a}$	$\mathbf{k_d}$
K	10^2 $M^{-1}s^{-1}$	10^{-2} s^{-1}	10^2 $M^{-1}s^{-1}$	10^{-2} s^{-1}	10^2 $M^{-1}s^{-1}$	10^{-2} s^{-1}	10^2 $M^{-1}s^{-1}$	10^{-2} s^{-1}
285.2	5.40 ± 0.02	10.71 ± 0.02	5.10 ± 0.04	12.95 ± 0.03	4.80 ± 0.03	14.15 ± 0.03	4.50 ± 0.03	14.82 ± 0.01
289.2	7.40 ± 0.02	12.64 ± 0.02	7.00 ± 0.04	14.85 ± 0.03	6.50 ± 0.03	15.89 ± 0.03	6.00 ± 0.03	16.81 ± 0.01
293.2	8.20 ± 0.05	14.63 ± 0.02	7.80 ± 0.05	16.95 ± 0.03	7.30 ± 0.04	18.08 ± 0.02	6.90 ± 0.01	18.84 ± 0.03
297.2	7.80 ± 0.04	17.05 ±0.01	7.50 ± 0.03	19.32 ± 0.02	7.10 ± 0.04	20.47 ± 0.02	6.70 ± 0.02	21.14 ± 0.02
298.2	7.50 ± 0.03	17.87 ±0 0.03	7.20 ± 0.03	19.96 ± 0.02	6.85 ± 0.03	21.06 ±0.02	6.50 ± 0.02	21.82 ± 0.02
301.2	6.30 ± 0.07	20.00 ± 0.02	6.10 ± 0.06	22.18 ± 0.05	5.80 ± 0.04	22.88 ± 0.04	5.60 ± 0.03	23.82 ± 0.03

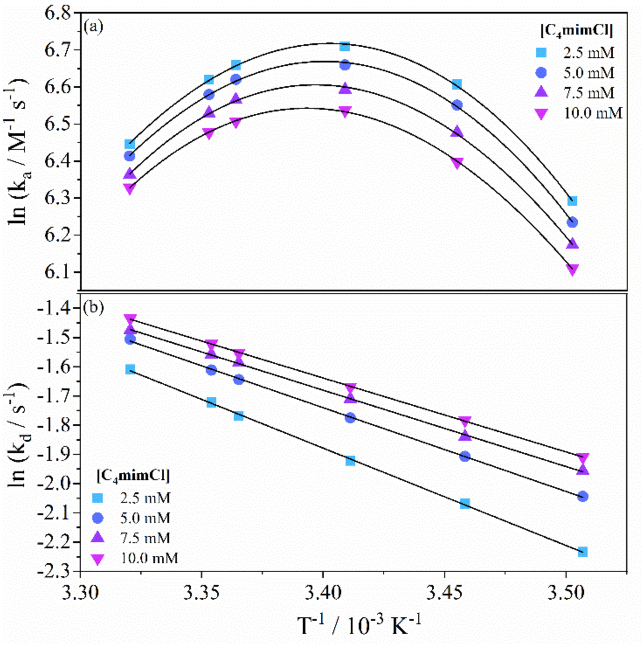

Fig. S7. Arrhenius plots of **(a)** ln k_a and **(b)** ln k_d associated with the interaction between SDBS and βCD−NH_2 as functions of reciprocal temperatures, in the presence of different C_4mimCl concentration.

Table S3. Energetic parameters for the formation of the activated complex [βCD–NH_2/SDBS]‡ in 2.5 mM of C_4mimCl at pH 7.4 and different temperatures.

**T**	**[C_4mimCl]**							
	2.5 mM							
	$\mathbf{E_a^‡}$	$\mathbf{\Delta H_a^‡}$	$\mathbf{\Delta G_a^‡}$	$\mathbf{T\Delta S_a^‡}$	$\mathbf{E_d^‡}$	$\mathbf{\Delta H_d^‡}$	$\mathbf{\Delta G_d^‡}$	$\mathbf{T\Delta S_d^‡}$
K	kJ mol^{-1}				kJ mol^{-1}			
285.2	70.40 ± 0.07	68.03 ± 0.07	54.81786 ± 0.00004	13.21 ± 0.02		25.333 ± 0.001	76.00548 ± 0.00004	-50.672 ± 0.001
289.2	36.02 ± 0.07	33.62 ± 0.07	54.8623 ± 0.00003	-21.24 ± 0.02		25.300 ± 0.001	76.60278 ± 0.00003	-51.303 ± 0.001
293.2	4.19 ± 0.07	1.75 ± 0.07	55.4051 ± 0.0001	-53.65 ± 0.02	27.704 ± 0.001	25.267 ± 0.001	77.18292 ± 0.00005	-51.916 ± 0.001
297.2	-25.26 ± 0.07	-27.74 ± 0.07	56.31810 ± 0.00005	-84.05 ± 0.02		25.234 ± 0.001	77.8115 ± 0.0001	-52.578 ± 0.001
298.2	-32.28 ± 0.07	-34.75 ± 0.07	56.61318 ± 0.00004	-91.36 ± 0.02		25.225 ± 0.001	77.9336 ± 0.0002	-52.708 ± 0.001
301.2	-52.50 ± 0.07	-55.00 ± 0.07	57.6445 ± 0.0001	-112.64 ± 0.02		25.200 ± 0.001	78.3260 ± 0.0002	-53.126 ± 0.001

Table S4. Energetic parameters for the formation of the activated complex [βCD–NH_2/SDBS]‡ in 5.0 mM of C_4mimCl at pH 7.4 and different temperatures.

**T**	**[C_4mimCl]**							
	5.0 mM							
	$\mathbf{E_a^‡}$	$\mathbf{\Delta H_a^‡}$	$\mathbf{\Delta G_a^‡}$	$\mathbf{T\Delta S_a^‡}$	$\mathbf{E_d^‡}$	$\mathbf{\Delta H_d^‡}$	$\mathbf{\Delta G_d^‡}$	$\mathbf{T\Delta S_d^‡}$
K	kJ mol^{-1}				kJ mol^{-1}			
285.2	70.1 ± 0.1	67.7 ± 0.1	54.95338 ± 0.00007	12.8 ± 0.1	23.832 ± 0.001	21.461 ± 0.001	74.58058 ± 0.00003	-53.120 ± 0.001
289.2	36.8 ± 0.1	34.3 ± 0.1	54.99642 ± 0.00005	-20.7 ± 0.1		21.427 ± 0.001	75.33113 ± 0.00002	-53.904 ± 0.001
293.2	5.5 ± 0.1	3.1 ± 0.1	55.5270 ± 0.0001	-52.4 ± 0.1		21.394 ± 0.001	76.08433 ± 0.00002	-54.690 ± 0.001
297.2	-23.6 ± 0.1	-26.1 ± 0.1	56.41500 ± 0.00004	-82.5 ± 0.1		21.361 ± 0.001	76.83264 ± 0.00001	-55.472 ± 0.001
298.2	-30.6 ± 0.1	-33.1 ± 0.1	56.71438 ± 0.00004	-89.8 ± 0.1		21.353 ± 0.001	77.01875 ± 0.00001	-55.666 ± 0.001
301.2	-50.9 ± 0.1	-53.4 ± 0.1	57.7252 ± 0.0001	-111.2 ± 0.1		21.328 ± 0.001	77.55472 ± 0.00002	-56.227 ± 0.001

Table S5. Energetic parameters for the formation of the activated complex [βCD–NH_2/SDBS]‡ in 7.5 mM of C_4mimCl at pH 7.4 and different temperatures.

T	[C_4mimCl]							
	7.5 mM							
	$E_a^{\ddagger}$	$\Delta H_a^{\ddagger}$	$\Delta G_a^{\ddagger}$	$T\Delta S_a^{\ddagger}$	$E_d^{\ddagger}$	$\Delta H_d^{\ddagger}$	$\Delta G_d^{\ddagger}$	$T\Delta S_d^{\ddagger}$
K	kJ mol^{-1}				kJ mol^{-1}			
285.2	64.91 ± 0.05	62.54 ± 0.05	55.09711 ± 0.00006	7.44 ± 0.05		19.355 ± 0.001	74.37048 ± 0.00002	-55.015 ± 0.001
289.2	36.78 ± 0.05	34.37 ± 0.05	55.17459 ± 0.00004	-20.80 ± 0.05		19.322 ± 0.001	75.16839 ± 0.00002	-55.846 ± 0.001
293.2	7.85 ± 0.05	5.41 ± 0.05	55.6884 ± 0.0001	-50.27 ± 0.05	21.726 ± 0.001	19.289 ± 0.001	75.92703 ± 0.00001	-56.638 ± 0.001
297.2	-21.77 ± 0.05	-24.24 ± 0.05	56.55041 ± 0.00005	-80.79 ± 0.05		19.256 ± 0.001	76.68979 ± 0.00001	-57.434 ± 0.001
298.2	-29.28 ± 0.05	-31.76 ± 0.05	56.83791 ± 0.00004	-88.59 ± 0.05		19.247 ± 0.001	76.88576 ± 0.00001	-57.638 ± 0.001
301.2	-52.01 ± 0.05	-54.51 ± 0.05	57.8515 ± 0.0001	-112.37 ± 0.05		19.222 ± 0.001	77.47692 ± 0.00002	-58.255 ± 0.001

Table S6. Energetic parameters for the formation of the activated complex [βCD–NH_2/SDBS]‡ in 10 mM of C_4mimCl at pH 7.4 and different temperatures.

**T**	**[C_4mimCl]**							
	10 mM							
	$\mathbf{E_a^{‡}}$	$\mathbf{\Delta H_a^{‡}}$	$\mathbf{\Delta G_a^{‡}}$	$\mathbf{T\Delta S_a^{‡}}$	$\mathbf{E_d^{‡}}$	$\mathbf{\Delta H_d^{‡}}$	$\mathbf{\Delta G_d^{‡}}$	$\mathbf{T\Delta S_d^{‡}}$
K	kJ mol^{-1}				kJ mol^{-1}			
285.2	62.31 ± 0.02	59.94 ± 0.02	55.25012 ± 0.00006	4.69 ± 0.02	21.028 ± 0.001	18.657 ± 0.001	74.26079 ± 0.00001	-55.603 ± 0.001
289.2	36.91 ± 0.02	34.50 ± 0.02	55.36702 ± 0.00005	-20.86 ± 0.02		18.624 ± 0.001	75.03308 ± 0.00001	-56.409 ± 0.001
293.2	9.77 ± 0.02	7.33 ± 0.02	55.82578 ± 0.00002	-48.49 ± 0.02		18.591 ± 0.001	75.82667 ± 0.00002	-57.236 ± 0.001
297.2	-18.93 ± 0.02	-21.40 ± 0.02	56.69367 ± 0.00003	-78.09 ± 0.02		18.558 ± 0.001	76.61022 ± 0.00001	-58.053 ± 0.001
298.2	-26.33 ± 0.02	-28.81 ± 0.02	56.96792 ± 0.00003	-85.78 ± 0.02		18.549 ± 0.001	76.79788 ± 0.00001	-58.249 ± 0.001
301.2	-49.04 ± 0.02	-51.54 ± 0.02	57.9394 ± 0.0001	-109.48 ± 0.02		18.524 ± 0.001	77.37610 ± 0.00002	-58.852 ± 0.001

Table S7. Thermodynamic parameters for the formation of the thermodynamically stable complex [βCD–NH_2/SDBS]° in 2.5 mM of C_4mimCl, at pH 7.4 and different temperatures.

**T**	**[C_4mimCl]**			
	2.5 mM			
	**K_b**	**ΔG°**	**ΔH°**	**TΔS°**
K	10^3 L mol^{-1}	kJ mol^{-1}		
285.2	5.0 ± 0.1	-20.2130 ± 0.0002	42.68 ± 0.07	62.91 ± 0.07
289.2	5.85 ± 0.09	-20.8557 ± 0.0001	8.31 ± 0.07	29.17 ± 0.07
293.2	5.60 ± 0.08	-21.0380 ± 0.0001	-23.53 ± 0.07	-2.48 ± 0.07
297.2	4.57 ± 0.02	-20.82335 ± 0.000005	-52.99 ± 0.07	-32.15 ± 0.07
298.2	4.20 ± 0.02	-20.67976 ± 0.000004	-59.99 ± 0.07	-39.30 ± 0.07
301.2	3.15 ± 0.04	-20.1693 ± 0.00	-80.22 ± 0.07	-60.03 ± 0.07

Table S8. Thermodynamic parameters for the formation of the thermodynamically stable complex [βCD–NH_2/SDBS]° in 5.0 mM of C_4mimCl, at pH 7.4 and different temperatures.

**T**	**[C_4mimCl]**			
	5.0 mM			
	**K_b**	**ΔG°**	**ΔH°**	**TΔS°**
K	10^3 L mol^{-1}	kJ mol^{-1}		
285.2	3.94 ± 0.03	-19.6272 ± 0.0001	46.3 ± 0.1	65.9 ± 0.1
289.2	4.71 ± 0.03	-20.3347 ± 0.0001	12.9 ± 0.1	33.3 ± 0.1
293.2	4.60 ± 0.03	-20.5574 ± 0.0001	-18.3 ± 0.1	2.3 ± 0.1
297.2	3.88 ± 0.02	-20.4174 ± 0.00004	-47.5 ± 0.1	-27.1 ± 0.1
298.2	3.61 ± 0.02	-20.30437 ± 0.00004	-54.5 ± 0.1	-34.2 ± 0.1
301.2	2.75 ± 0.03	-19.8295 ± 0.0001	-74.8 ± 0.1	-54.9 ± 0.1

Table S9. Thermodynamic parameters for the formation of the thermodynamically stable complex [βCD–NH_2/SDBS]° in 7.5 mM of C_4mimCl, at pH 7.4 and different temperatures.

**T**	**[C_4mimCl]**			
	7.5 mM			
	**K_b**	**ΔG°**	**ΔH°**	**TΔS°**
K	10^3 L mol^{-1}	kJ mol^{-1}		
285.2	3.39 ± 0.02	-19.2734 ± 0.0001	43.18 ± 0.05	62.46 ± 0.05
289.2	4.09 ± 0.02	-19.99381 ± 0.00005	15.05 ± 0.05	35.04 ± 0.05
293.2	4.04 ± 0.02	-20.2386 ± 0.0001	-13.87 ± 0.05	6.36 ± 0.05
297.2	3.47 ± 0.02	-20.13938 ± 0.00005	-43.50 ± 0.05	-23.36 ± 0.05
298.2	3.25 ± 0.01	-20.04785 ± 0.00004	-51.00 ± 0.05	-30.96 ± 0.05
301.2	2.53 ± 0.02	-19.6254 ± 0.0001	-73.74 ± 0.05	-54.11 ± 0.05

Table S10. Thermodynamic parameters for the formation of the thermodynamically stable complex [βCD–NH_2/SDBS]° in 10 mM of C_4mimCl, at pH 7.4 and different temperatures.

**T**	**[C_4mimCl]**			
	10 mM			
	**K_b**	**ΔG°**	**ΔH°**	**TΔS°**
K	10^3 L mol^{-1}	kJ mol^{-1}		
285.2	3.04 ± 0.02	-19.0107 ± 0.0001	41.29 ± 0.02	60.30 ± 0.02
289.2	3.57 ± 0.02	-19.66606 ± 0.00005	15.88 ± 0.02	35.55 ± 0.02
293.2	3.66 ± 0.01	-20.00088 ± 0.00003	-11.26 ± 0.02	8.75 ± 0.02
297.2	3.17 ± 0.01	-19.91654 ± 0.00003	-29.96 ± 0.02	-20.04 ± 0.02
298.2	2.98 ± 0.01	-19.82996 ± 0.00003	-47.36 ± 0.02	-27.53 ± 0.02
301.2	2.35 ± 0.01	-19.4367 ± 0.0001	-70.07 ± 0.02	-50.63 ± 0.02
